# Seroprevalence of select bloodborne pathogens and associated risk behaviors among injection drug users in the Paso del Norte region of the United States – Mexico border

**DOI:** 10.1186/1477-7517-5-33

**Published:** 2008-11-16

**Authors:** Joan P Baumbach, Lily N Foster, Mark Mueller, Michelle Firestone Cruz, Sonia Arbona, Sharon Melville, Rebeca Ramos, Steffanie A Strathdee

**Affiliations:** 1 New Mexico Department of Health, Santa Fe, NM, USA; 2 Centre for Addiction and Mental Health, Toronto, ON, Canada; 3 Texas Department of State Health Services, Austin, TX, USA; 4 United States – Mexico Border Health Association, El Paso, TX, USA; 5 University of California, San Diego, CA, USA

## Abstract

**Background:**

The region situated where the borders of Mexico, Texas and New Mexico meet is known as 'Paso del Norte'. The Paso del Norte Collaborative was formed to study the seroprevalence of select pathogens and associated risk behaviors among injection drug users (IDUs) in the region.

**Methods:**

Respondent-driven sampling (RDS) was used: 459 IDU participants included 204 from Mexico; 155 from Texas; and 100 from New Mexico. Each of the three sites used a standardized questionnaire that was verbally administered and testing was performed for select bloodborne infections.

**Results:**

Participants were mostly male (87.4%) and Hispanic/Latino (84.7%) whose median age was 38. In Mexico, Texas and New Mexico, respectively: hepatitis B virus (HBV) was seen in 88.3%, 48.6% and 59.6% of participants; hepatitis C virus (HCV) in 98.7%, 76.4% and 80.0%; human immunodeficiency virus (HIV) in 2.1%, 10.0% and 1.0%; and syphilis in 4.0%, 9.9% and 3.0%. Heroin was the drug injected most often. More IDUs in New Mexico were aware of and used needle exchange programs compared with Texas and Mexico.

**Conclusion:**

There was mixed success using RDS: it was more successfully applied after establishing good working relationships with IDU populations. Study findings included similarities and distinctions between the three sites that will be used to inform prevention interventions.

## Introduction

**Figure 1:**
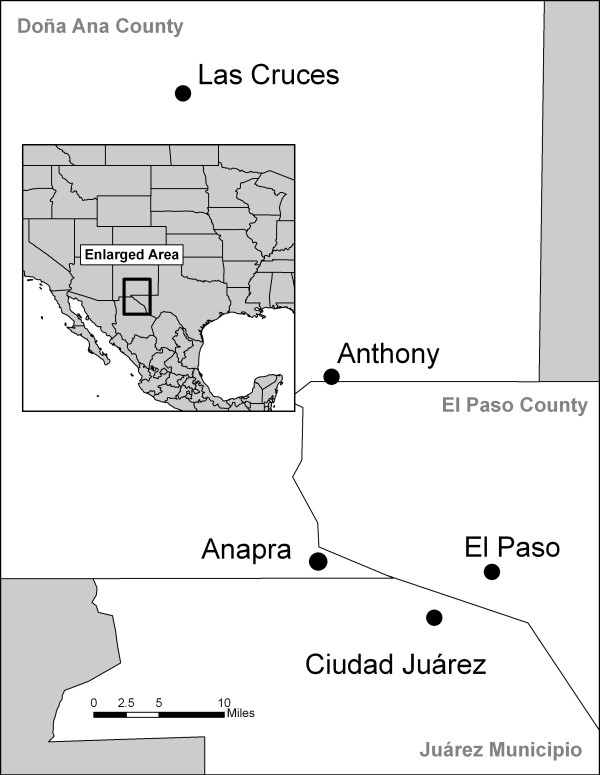
Area map of Paso del Norte Region.

The 'Paso del Norte' region (See Figure 1) straddles the midpoint of the U.S-Mexico border. It contains Ciudad Juárez in Chihuahua, Mexico, El Paso, Texas and Doña Ana County, New Mexico. It is a suitable site for the study of risk behaviors associated with injection drug use because: a) many people there lead lives on both sides of the border; b) there are known injection drug use (IDU) populations in the three separate states involved; c) the populations in these states are managed by different programs and policies.

Ciudad Juárez had an estimated population of 1.2 million people in 2000 [1], 60% of which originate from other parts of Mexico. The transient population is estimated at 250,000 persons [2,3]. After Tijuana in Baja California, Ciudad Juárez is thought to have the largest population of illicit drug users in Mexico, estimated to be twice that of the national average [4]. In 2001, a community-based survey found that there were approximately 6,000 IDUs in Juárez [5]. According to the U.S. Census Bureau, El Paso County in Texas had 679,622 inhabitants in 2000 of which 563,662 lived in the city of El Paso. The Texas Department of State Health Services (DSHS) is an agency of the Texas Health and Human Services System whose mission is to improve health and well-being in Texas: one statewide indicator is that 26% of the 88,452 admissions to DSHS-funded treatment programs in 2007 had a history of IDU [6]. In 2000, according to the U.S. Census Bureau, 174,682 persons lived in Doña Ana County, New Mexico, of whom 74,267 lived in Las Cruces. The estimated IDU population for 2003 was approximately 1,400. This estimate was derived from the National Survey on Drug Use and Health and local indicator data (i.e., rates of drug-related death and infectious disease incidence) (N. Shah; New Mexico Department of Health, written communication; October 2005). There have been relatively few studies of bloodborne infections among IDUs in the Paso del Norte region. However, in 2006, in the jurisdiction of Juárez, El Paso County and Doña Ana County, newly reported rates of human immunodeficiency virus (HIV) among all risk groups were 6.5 (G Barrios Gallegos, State Epidemiologist, Chihuahua, Mexico, written communication, September, 2007), 8.0 [7] and 2.5 per 100,000 (K Rooney, Epidemiologist, New Mexico Department of Health, written communication; September, 2007), respectively. In a series of seroprevalence studies using convenience sampling conducted in Texas, hepatitis C virus (HCV) seroprevalence for IDUs entering drug treatment centers was 84.5%; 15% for IDUs tested from sexually transmitted disease (STD) clinics, and 29.2% for IDUs at HIV testing sites [8]. Data from HIV counseling and testing sites in El Paso revealed that 2.8% of IDUs in those settings were positive for HIV between 2003 and 2006 (J Hitt; Texas DSHS HIV Prevention Program Counseling and Testing Data, written communication; September, 2007). Seroprevalence results obtained during studies of convenience samples of IDUs in New Mexico in 1995 and 1997 revealed high rates of antibody positive for HCV (82.2%) and hepatitis B virus (HBV) (61.1%) and a low rate for HIV (0.5%): 90% of the IDUs reported sharing injection equipment, 52% with friends and 30.9% with their main sex partner [9]. While these studies demonstrated associations between infection with HBV and HCV and certain risk behaviors, methodological limitations of convenience sampling were noted [10].

The question of how to reduce the risk of bloodborne disease transmission among IDUs in this region raises interesting issues for consideration. Harm reduction is a fairly new concept in Mexico and one that has often been met with controversy by government. The first needle exchange program in Mexico started in the 1980s in Juárez, Chihuahua and is operated by Programa Compañeros, A.C. [11,12], a non-governmental organization that began in 1986 to assist individuals living with HIV/AIDS and drug addictions: Programa Compañeros provides a number of prevention, treatment and social services, including street and prison-based harm reduction programs. The second needle exchange program in Mexico opened in Tijuana, Baja California, in 2004 and is operated by Prevencasa A.C. In addition, there are currently small-scale needle exchange programs operating in five other states: Coahuila, Nuevo Leon, Oaxaca, Sinaloa and Zacatecas [13]. In El Paso County, Texas, DSHS has provided substance abuse treatment since 1980 through its treatment center, Aliviane. In addition it provides prevention education, counseling, testing for HIV and referrals for treatment of infectious diseases and other health problems. The seroprevalence and risk behavior results of New Mexico studies from the mid 1990s [9] were used to support the successful implementation by the New Mexico Department of Health of a syringe exchange policy enacted by the New Mexico Legislature in 1998. Since then, over 10,000 individuals have enrolled in the Syringe Exchange Program with nearly seven million syringes collected and distributed statewide (B. Lieving, New Mexico Department of Health, written communication; May, 2007).

The Paso del Norte Collaborative, comprised of governmental public health workers, health services providers, non-governmental organizations and academicians, was formed in order to learn more about the IDU population in the region. This paper focuses on the seroprevalence of bloodborne infectious diseases and select associated risk behaviors derived from the Paso del Norte Collaborative study.

## Methods

### Respondent-driven sampling

The study was designed to be representative of the general IDU population in the Paso del Norte region. To that end, respondent driven sampling (RDS) was used. RDS is a modified form of chain-referral sampling that relies on members of a hidden population, such as IDUs, to recruit their peers. Since its initial implementation to study IDUs in several small Connecticut cities [14], RDS has been evaluated and proven an effective way to study hidden populations, particularly those that are at risk for HIV infection [15-19]. RDS has been used in several settings that include large urban centers [20,21], smaller cities [14,22], and mostly non-urban areas [23]. RDS attempts to eliminate sampling bias by selection of initial respondents called 'seeds' who have diverse gender, race/ethnicity, and drug preferences. This is an attempt to reach what is termed homophily (i.e., the tendency for an individual seed to recruit persons who are similar) in different recruitment waves, each of which start from the seeds who were recruited for their specific characteristics: the goal is to arrive at a representative sample. However, limitations in this sampling method certainly can include biases, particularly those that might lead to a more homogeneous group of participants ultimately recruited than a truly representative sample of the general IDU population in a given geographic area.

In this study, a diverse group of seeds who were current IDUs were recruited to initiate the process: they were heterogeneous in age, gender, race/ethnicity and drug/s of choice. Each seed was enrolled into the study and provided with three uniquely coded coupons to refer to their peers. Each peer enrolled into the study was also provided three uniquely coded coupons. In all three sites, participants received modest monetary reimbursement for their own enrollment into the study and if they returned for laboratory results. In New Mexico and Texas, participants received additional modest monetary reimbursement for each recruit successfully enrolled into the study. RDS was used to recruit 205 and 155 subjects in Juárez and El Paso, respectively, while New Mexico recruited 83 subjects by RDS and 17 were a convenience sample.

### Study population and sites

In each study site, participants eligible for the study were: a) 18 years or older; b) English or Spanish speaking; c) active IDUs defined as having had at least one injection in the past thirty days as demonstrated by injection stigmata (i.e. skin 'track' marks) or clear ability to describe injection drug methods and habits; d) willing and able to provide written informed consent; and e) not previously interviewed for the study. Study participants were not aware of the eligibility requirements so as not to bias ongoing recruitment. Data collection occurred between February-March 2005 in Ciudad Juárez, January-March 2005 in Doña Ana County and February-August 2006 in El Paso.

### Enrollment and questionnaire

The study process included: a) a screening using specific inclusion criteria; b) a blood draw; c) a questionnaire administered verbally in English or Spanish; d) medical oversight by licensed physicians; e) exit procedures that included provision of prevention information, referrals to services and distribution of coupons to be given to subsequent recruits from their social network; f) return of participants for laboratory results. Participants at the New Mexico site were able to receive hepatitis A and/or B vaccination if indicated by laboratory results. Hepatitis A and B vaccines were available to all participants enrolled in the Texas site. Study methods were approved by the Institutional Review Boards of the three respective areas.

Each of the three sites used a standardized questionnaire that was verbally administered by trained study workers. Most questions had been previously validated: those that had not were piloted in English and Spanish prior to implementation of the study. New Mexico and Texas used identical questionnaires which represented a subset of the questions used in Mexico. Cultural sensitivity and nonjudgmental approaches were stressed during training of the study workers and throughout the study. The survey included questions on: a) demographics; b) social network size and relationship to the person from whom coupon was received (these questions are needed for RDS calculations); c) socioeconomics (e.g., marital status, living sites, average monthly income, sources of income, ever incarcerated); d) injection drug use and needle sharing behavior; e) sexual behavior; f) infectious disease history; g) health services and insurance coverage.

### Laboratory testing

The New Mexico Department of Health Scientific Laboratory Division (SLD) conducted all testing for the New Mexico and Mexico participants who supplied sufficient quantity of serum. Testing for hepatitis A virus (HAV) was performed on specimens collected in New Mexico but not in Mexico: in addition, both sites had tests for HIV-1, HBV, HCV and syphilis. Specimens collected in Texas were tested by the Texas DSHS laboratory in Austin, Texas. A few specimens were tested for HIV-1 at the El Paso City-County Health and Environmental District Laboratory. All Centers for Disease Control and Prevention (CDC) permits and customs documents were completed according to government standards. Confirmed HIV positive specimens from Mexico were also sent to the Institute of Human Virology, University of Maryland Biotechnology Institute for viral sequencing.

For New Mexico specimens, HIV-1 antibody testing was conducted using an enzyme-linked immunosorbent assay, the Vironostika HIV-1 Microelisa System (bioMerieux, Inc., Durham, NC). Specimens that were repeatedly reactive were further tested by the Genetic Systems HIV-1 Western Blot assay (Bio-Rad Laboratories, Inc., Hercules, CA). For Mexico specimens, a drop of blood from each sample was used in the Determine^® ^HIV-1/2 Test (Abbott Laboratories, Kampala, Uganda); this rapid test is approved for developing countries. The remainder of clinical specimens from Mexico was sent to DSHS in El Paso and from there to SLD for the remainder of testing. Hepatitis testing was conducted using a number of enzyme immunoassays: for HAV, ETI-AB-HAVK PLUS (DiaSorin, Inc., Stillwater, MN); for hepatitis B core antibody (anti-HBc), ETI-AB-COREK PLUS (DiaSorin, Inc., Stillwater, MN); for hepatitis B surface antigen (HBsAg), Genetic Systems HBsAg EIA 3.0 (Bio-Rad Laboratories, Inc., Hercules, CA); for HCV, Ortho HCV Version 3.0 ELISA (Ortho-Clinical Diagnostics, Rochester, NY). Syphilis screening was done using the Impact Rapid Plasma Reagin (RPR) Card Test (Inverness Medical, Princeton, NJ) and confirmed by a *Treponema pallidum *particle agglutination (TP PA) test, the Serodia-TP PA kit (Fujirebio Diagnostics, Inc., Malvern, PA). With the exception of the tests for HBsAg and initial syphilis screening, the DSHS laboratory used the same tests as SLD. Testing for HBsAg was done using ETI-MAK-2 PLUS (DiaSorin, Inc., Stillwater, MN). Screening for syphilis was conducted by the ASI RPR 18 mm Card Test (Arlington Scientific, Inc., Springville, UT); reactive specimens were confirmed by the Serodia-TP PA kit (Fujirebio Diagnostics, Inc., Malvern, PA).

Given the requirements for anonymity and the highly mobile nature of the participants, care was taken to advise participants in the study of when and where to return for their laboratory results. In order to obtain results, participants provided key information that linked to the unique codes submitted with their clinical specimens.

### Statistical analysis

Demographics of the study population, rates of select bloodborne infections (i.e., HAV, HBV, HCV, HIV and syphilis), and some associated risk behaviors were analyzed to provide a basic description of this IDU population. Data was collected on site using Microsoft Excel (Microsoft, Redmond, WA) and later merged and analyzed using SAS version 9.1 (SAS Institute Inc, Cary, NC).

All categorical variables – including sociodemographic and risk behavior information – were compared across the three sites using the Chi-square test of independence. In the case of small numbers, Fisher's Exact method was employed to detect significant differences across sites. The continuous variables of age, years of education, social network size and days since last injection were compared across sites using one-way analysis of variance (ANOVA) methods.

Crude seroprevalence was calculated by observing the number of blood samples that tested positive out of the total number of samples that could be processed given adequate quantity and quality of clinical specimens. RDS seroprevalence adjustments for the Texas and Mexico data were calculated using the RDS Analysis Tool v.5.6.0 (Cornell University, 2003). New Mexico was unable to utilize RDS analytic tools because of the number of study participants who were recruited as seeds or convenience samples and also because recruitment patterns were not heterologous enough for a number of traits (e.g., those who were hepatitis A positive only recruited others who happened to be hepatitis A positive). Without more cross-recruitment, RDS adjustments cannot be calculated [24].

## Results

### Demographic and social characteristics

Of the 459 study participants, 204 were from Ciudad Juárez, 155 from El Paso and 100 from Doña Ana County. Overall, 401 (87.4%) were male and 58 (12.6%) were female (Table 1). The median age was 38 years (33 years in Mexico and 42 years in both Texas and New Mexico). Information was available from 456 respondents on ethnicity: 389 (84.7%) identified as Hispanic/Latino and 67 (14.6%) as not Hispanic/Latino. Education status was expressed as number of years of education completed and the overall median was 9 years: 7 in Mexico, 11 in Texas and 11.5 in New Mexico, respectively.

**Table 1:** Demographic and social characteristics of study participants

	Doña Ana County, NM (n = 100)	Ciudad Juárez, MX (n = 204)	El Paso, TX (n = 155)	Chi-square p-value^a^	All sites (n = 459)
Sex, n (%)					
Male	80 (80.0)	189 (92.7)	132 (85.2)		401 (87.4)
Female	20 (20.0)	15 (7.4)	23 (14.8)	0.005	58 (12.6)
Age. mean (median)	41.8 (42.0)	35.3 (33.0)	42.0 (42.0)	< 0.0001^b^	39.0 (38.0)
Hispanic, n (%)					
Yes	69 (69.0)	203 (99.5)	117 (75.5)		389 (84.7)
No	31 (31.0)	1 (0.5)	35 (22.5)	< 0.0001	67 (14.6)
No answer	0 (0.0)	0 (0.0)	3 (1.9)		3 (0.7)
Country of birth, n (%)					
USA	94 (94.0)	4 (2.0)	139 (89.7)		237 (51.6)
Mexico	4 (4.0)	200 (98.0)	14 (9.0)		218 (47.5)
Other	1 (1.0)	0 (0.0)	1 (0.6)		2 (0.4)
No answer	1 (1.0)	0 (0.0)	1 (0.6)		2 (0.4)
Years of education, mean (median)	10.9 (11.5)	7.1 (7.0)	10.8 (11)	< 0.0001^b^	9.2 (9.0)
Marital status, n (%)					
Unmarried	41 (41.0)	105 (51.5)	66 (42.6)		212 (46.2)
Married	27 (27.0)	62 (30.4)	36 (23.2)		125 (27.2)
Divorced/Separated/Widowed	32 (32.0)	36 (17.6)	52 (33.5)	0.007	120 (26.1)
No answer	0 (0.0)	1 (0.5)	1 (0.6)		2 (0.4)
Social network, mean (median)	21.0 (10.0)	52.2 (20.0)	28.5 (15.0)	0.002^b^	37.4 (20.0)
Days since last injection, mean (median)	5.2 (2.0)	0.8 (0.0)	3.7 (1.0)	< 0.0001^b^	2.7 (1.0)
Family ever inject drugs*, n (%)					
None	49 (49.0)	118 (57.8)	84 (54.2)		251 (54.7)
Mother/father	10 (10.0)	12 (5.9)	13 (8.4)		35 (7.6)
Sibling	40 (40.0)	49 (24.0)	51 (32.9)		140 (30.5)
Cousin	12 (12.0)	17 (8.3)	18 (11.6)		47 (10.2)
Aunt/uncle	17 (17.0)	15 (3.3)	22 (14.2)		54 (11.8)
Other	11 (11.0)	24 (5.2)	20 (12.9)		55 (12.0)
Don't know	2 (2.0)	2 (0.4)	4 (2.6)		8 (1.7)
Ever have tattoo					
Yes	80 (80.0)	167 (81.9)	119 (76.8)		366 (79.7)
No	20 (20.0)	37 (18.1)	33 (21.3)	0.702	90 (19.6)
No answer	0 (0.0)	0 (0.0)	3 (1.9)		3 (0.7)

*Categories are not mutually exclusive and therefore percentages may be greater than 100%.

^a ^Chi-square test of independence used to observe differences in categorical variables across sites.

^b ^ANOVA used to compare means across sites.

Median social network size ("How many people do you know by name or street name in the past six months who also shoot up?") was 20: median sizes were 20 in Mexico, 14 in Texas and 10 in New Mexico. Only 251 (54.7%) participants overall reported no family members ever injecting drugs.

### Seroprevalence

Testing for HAV was conducted in New Mexico and Texas. Using RDS, 73% of Texas IDUs had evidence of HAV compared with New Mexico's crude finding among 66% of participants (Table 2). Hepatitis B testing was done at all 3 study locations: RDS calculations revealed that Mexico had 88.3% and Texas 48.6% of IDUs positive for anti-HBc, revealing individuals who were not susceptible to infection (i.e., immune due to natural infection whether acute infection or chronically infected), compared with New Mexico's crude calculation of 59.6%. RDS calculations for HCV revealed that 98.7% screened positive in Mexico and 76.4% in Texas compared with New Mexico's crude calculation of 80.0%. RDS calculations for proportion of individuals who were HIV-positive revealed 2.1% for Mexico, 10.0% for Texas and a crude 1.0% for New Mexico. Syphilis was seen among 4.0% of participants in Mexico and 9.9% in Texas using RDS calculations compared with New Mexico's crude calculation of 3.0%.

**Table 2:** Crude and RDS-adjusted seroprevalence of infectious diseases among study participants

	Doña Ana County, NM				Ciudad Juárez, MX				El Paso, TX				Crude % p-value
	
	Positive	Total*	Crude %	RDS %	Positive	Total	Crude %	RDS %	Positive	Total	Crude %	RDS %	
HAV	66	97	68.0	-	-	-	-	-	105	148	70.9	73.0	0.740 ^a^
HBV	56	94	59.6	-	171	203	84.2	88.3	74	137	54.8	48.6	< .0001 ^b^
HCV	76	95	80.0	-	194	203	95.6	98.7	122	147	83.0	76.4	< .0001^a^
HIV	1	100	1.0	-	6	203	3.0	2.1	9	155	5.8	10.0	0.109^b^
Syphilis	3	100	3.0	-	7	193	3.6	4.0	8	150	5.3	9.9	0.605^a^

*Total excludes participants whose sample was either insufficient or rejected for testing.

^a ^Chi-square test of independence used to observe differences among crude seroprevalence across sites.

^b ^Fisher's Exact method used to run test of independence due to > 20% of cells with less than 5 observations.

### Risk behaviors

Injection drug habits were different between sites with a higher percent (97.1%) of respondents in Mexico reporting injecting at least daily in the past six months compared with 64.5% and 55.0% in Texas and New Mexico, respectively (p < 0.0001) (Table 3). Heroin alone was the drug injected most often in the past six months in New Mexico (69.0%) and Texas (66.5%), whereas in Mexico the 'speedball' or combination of heroin and cocaine was the most frequently injected (41.3%) in the past six months (p < 0.0001). More respondents in Mexico (64.7%) and Texas (62.6%) reported using previously used needles in the past six months compared with New Mexico respondents (38.0%) (p < 0.0001). More respondents in New Mexico (71.0%) were aware of needle exchange programs in their area compared with Mexico (14.2%) and Texas (11.0%) (p < 0.0001). Numbers of participants who answered questions about awareness and use of needle exchange programs in the previous six months were relatively low. Of all sites combined, 57.7% (71/123) reported being aware of needle exchange and had used a program in the past six months: 68.4% (52/76) in Doña Ana County; 58.6% (17/29) in Ciudad Juárez; 11.1% (2/18) in El Paso.

**Table 3:** Risk behaviors among study participants

	Doña Ana County, NM (n = 100)	Ciudad Juárez, MX (n = 204)	El Paso, TX (n = 155)	Chi-square p-value ^a^	All sites (n = 459)
In the past 6 months, how often did you inject drugs?					
Less than daily	43 (43.0)	5 (2.5)	49 (31.6)		97 (21.1)
Several times a day	55 (55.0)	198 (97.1)	100 (64.5)	< 0.0001	353 (76.9)
No answer	2 (2.0)	1 (0.5)	6 (3.9)		9 (2.0)
In the past 6 months, how often have you used a needle that you knew had been used before?					
Never	60 (60.0)	71 (34.8)	14 (9.0)		145 (31.6)
Share	38 (38.0)	132 (64.7)	97 (62.6)	< 0.0001	267 (58.2)
No answer	2 (2.0)	1 (0.5)	44 (28.4)		47 (10.2)
Are you aware of any needle exchange programs in your area?					
No	29 (29.0)	174 (85.3)	135 (87.1)		338 (73.6)
Yes	71 (71.0)	29 (14.2)	17 (11.0)	< 0.0001	117 (25.5)
No answer	0 (0.0)	1 (0.5)	3 (1.9)		4 (0.9)
Which of the following drugs do you inject most often in the past 6 months?					
Heroin	69 (69.0)	82 (40.2)	103 (66.5)		254 (55.3)
Cocaine	17 (17.0)	4 (2.0)	29 (18.7)		50 (10.9)
Speedball (heroin + cocaine)	2 (2.0)	84 (41.2)	14 (9.0)		100 (21.8)
Other	5 (5.0)	10 (4.9)	6 (3.9)	< 0.0001	21 (4.6)
No answer	7 (7.0)	24 (11.8)	3 (1.9)		34 (7.4)

^a ^Chi-square test of independence used to observe differences across sites.

## Discussion

These findings from the Paso del Norte Collaborative study describe a group of IDUs who are mainly Hispanic/Latino, male (87.4%), with a median age of 38, relatively poorly educated (median years of education 9), who have relatively high rates of infectious hepatitis and much lower rates of HIV and syphilis. The median age and gender characteristics of the IDU population in this study were similar to those seen in other studies of seroprevalence among IDUs [25,26]. While the demographics of the IDU population studied were similar, some findings were site-specific: a) mean social network size of Mexico participants was larger than in Texas, which in turn was larger than in New Mexico; b) participants from Mexico injected more frequently than those from Texas or New Mexico; c) while Texas had much lower rates of HBV and HCV than Mexico and slightly lower rates than New Mexico, they had higher rates of HIV and syphilis. The common and site-specific findings in this study will enable those working in the region to design and implement services for their populations more precisely.

New Mexico participants reported being aware of and using needle exchange programs in their area more than reported at the other two sites. Overall, there were high rates of injection drug use seen in families, including inter-generational findings. The Mexico site reported larger social network sizes and more frequent injection drug use behavior, factors which have been studied in the context of transmission of HBV, HCV and HIV [27-30]. The higher rates of HIV and syphilis among Texas residents compared with Mexico and New Mexico were not matched by their rates of HBV and HCV, both of which were much lower than those of Mexico and slightly lower than New Mexico.

There were a number of limitations of the study. The total number of study participants was less than optimal. New Mexico recruited fewer than 10% of the projected IDU population in Doña Ana County. Texas and New Mexico used significantly more seeds than Mexico. New Mexico enrolled 17% of participants as a convenience sample because of slow enrollment. In addition, for a number of traits among New Mexico participants, recruitment patterns were insular. These limitations in the New Mexico sample precluded RDS analysis thereby making a tri-state binational aggregated RDS analysis impossible. Although Texas recruited a similar number of seeds as New Mexico, their sample characteristics allowed for RDS calculations. Another limitation is that data collection did not occur during the same time frame for the three sites.

One of the strengths of the Paso del Norte Collaborative study was the unified approach toward the development of methods, training of study workers, implementation of the study and analysis of data. All three sites contributed to decision-making. Individuals from the three study sites visited each other as training was being conducted for study workers and again as the study was rolled out in phases: lessons learned were shared throughout the study. The same database shell was used for on-site management of questionnaires completed, coupons distributed, and distribution of laboratory results and modest monetary reimbursements. Representatives from each site performed quality control of data. Data-sharing agreements were formalized which include review of quality and interpretation of data by named representatives from all three sites.

## Conclusion

This first report of select findings from the Paso del Norte Collaborative study provides baseline information that can help local public health systems develop programs and policies aimed at reducing the harms associated with injection drug use and contribute to future studies to prevent and control injection drug use and related transmission of bloodborne pathogens in this region. Although, in general, there was mixed success using RDS, in Mexico, where a non-governmental organization had achieved longstanding trust in the IDU community, RDS worked very well compared to governmental efforts in New Mexico. Any future studies of this kind would benefit from the lessons learned by the Paso del Norte Collaborative. These lessons include: a) formalized collaboration among study workers and with study participants is useful at all stages of the study; b) RDS is more successfully applied after establishing good working relationships with the IDU population. While some common threads are seen in the study findings, there are also distinctions between the three states and the two countries that could benefit from further analysis and study. It will be important to continue to follow seroprevalence and risk behavior data over time to monitor the impact of services, programs and policies in the Paso del Norte region, including the statewide syringe exchange services provided by the New Mexico Department of Health Harm Reduction Program.

## Competing interests

The authors declare that they have no competing interests.

## Authors' contributions

JB helped design the study, supervise and assist with aspects of implementation, synthesized analyses, and led the writing. LF helped design the study, assist with implementation, complete analyses and assist with writing. MM helped design the study, assist with implementation, complete analyses and assist with writing. MFC helped design the study, assist with implementation and interpretation of findings. SA supervised implementation and helped with interpretation of findings. SM helped design the study and supervise aspects of implementation. RR helped design the study, with data collection and interpretation of findings. SS helped design the study and supervise implementation. All authors read and approved the final manuscript.
